# A standardized surgical technique for debridement, antibiotics and implant retention improves treatment success rates in acute periprosthetic knee infection

**DOI:** 10.1002/ksa.70350

**Published:** 2026-02-24

**Authors:** Guillermo Sánchez‐Rosenberg, Anna Bartsch, Mario Morgenstern, Richard Kuehl, Martin Clauss

**Affiliations:** ^1^ Center for Musculoskeletal Infections (ZMSI) University Hospital Basel Basel Switzerland; ^2^ Department of Orthopedics and Trauma Surgery University Hospital Basel Basel Switzerland; ^3^ Department of Infectious Diseases University Hospital Basel Basel Switzerland

**Keywords:** acute PJI, debridement, antibiotics and implant retention (DAIR), total knee arthroplasty (TKA), periprosthetic joint infection (PJI), standardized surgical protocol

## Abstract

**Purpose:**

Results after debridement, antibiotics and implant retention (DAIR) for acute periprosthetic knee joint infections (PJIs) vary widely. Although data are limited, surgeon expertise and surgical technique likely influence outcomes. Standardized DAIR performed by surgeons within a multidisciplinary team (MDT) dedicated to the treatment of PJI was hypothesized to improve treatment success.

**Methods:**

Patients undergoing DAIR for acute knee PJI at our institution were retrospectively identified, and the outcomes between the two temporally distinct cohorts were compared. Patients treated between January 2005 and May 2015 without a standardized surgical DAIR technique or MDT support comprised the non‐standardized cohort. The standardized cohort comprised of patients treated between October 2019 and October 2022 with a standardized DAIR technique and the involvement of an MDT. Treatment success was defined according to the Delphi consensus criteria.

**Results:**

After screening for eligibility and applying exclusion criteria, 26 cases from the standardized cohort were compared with 31 from the non‐standardized cohort. A higher successful treatment rate treatment success rate of 96.3% was observed in the standardized cohort, when compared to the 80.6% success rate of the non‐standardized cohort (*p* = 0.12). Patients in the standardized cohort were older (standardized 75.5 ± 11.2; non‐standardized 69.2 ± 9; *p* = 0.02) and had a longer median interval from time of implantation to DAIR (median interquartile range [IQR] standardized 2.5–13.5 years, non‐standardized 0.4–2.8 years; *p* = 0.98). With respect to the preoperative laboratory values, no significant differences were observed, except for the percentage of polymorph nuclear cells in synovial fluid (standardized 86.3%, non‐standardized 90.6%; *p* = 0.02). *Staphylococcus aureus* was the most frequently isolated pathogen in both cohorts (standardized [32%], non‐standardized cohort [24.13%]; *p* = 0.55).

**Conclusion:**

Performing DAIR using a standardized surgical technique with a dedicated MDT resulted in higher treatment success rates when compared to a non‐standardized approach.

**Level of Evidence:**

Level III retrospective comparative study.

**Abbreviations**
DAIR: debridement, antibiotics and implant retention
EBJIS: European Bone and Joint Infection Society
ID: infectious disease
IQR: interquartile range
MDT: multidisciplinary team
PE: polyethylene
PJI: periprosthetic joint infection
TKA: total knee arthroplasty

## INTRODUCTION

With an incidence ranging between 1% and 2%, periprosthetic joint infection (PJI) is a serious complication after total knee arthroplasty (TKA) [25]. Appropriate management begins with adequate diagnosis as per the European Bone and Joint Infection Society (EBJIS) definition [30], followed by standardized surgical intervention and organism‐targeted antibiotic treatment [11, 36].

Debridement, antibiotics and implant retention (DAIR) is currently the surgical intervention of choice for acute PJI. Acute PJI includes early postoperative infections with an onset of less than 4–6 weeks after surgery or acute hematogenous infections with symptom duration of <3 weeks [11, 36, 46]. DAIR is an attractive surgical option in comparison to one or two‐stage prosthetic exchanges. In addition to being technically less demanding, it is also biologically favourable due to a lesser degree of invasiveness, which allows bone stock preservation and better post‐operative joint function [3, 14, 21].

However, clinical success rates after DAIR vary widely, ranging from 16% to 88% [2, 5, 8, 10, 11, 23, 24, 27, 28, 29, 34, 39]. Older age, severity of presenting illness and symptoms, comorbidities, virulent pathogens, and failure to exchange removable components are well‐studied risk factors associated with worse outcomes [36, 45]. Although rarely addressed in the literature, available evidence suggests that surgeon experience and the performance of an exhaustive surgical and mechanical debridement following a standardized protocol play a key role in improving treatment success rates after DAIR [2, 11, 17, 19, 20, 34, 35, 41].

The purpose of this study was to evaluate the effect of a standardized DAIR technique on the treatment success rates in acute PJI after TKA when performed by surgeons belonging to a multidisciplinary team (MDT) focusing solely on the management of musculoskeletal infection. To address the current gap in the literature regarding the true impact of a standardized DAIR technique on clinical outcomes of acute PJI after TKA, it was hypothesized that a standardized DAIR performed by experienced surgeons within an MDT dedicated to the treatment of PJI would improve treatment success when compared to a non‐standardized approach.

## METHODS

The treatment success between two cohorts of patients who underwent DAIR for suspected acute PJI after TKA was compared. Although both cohorts were treated in different time periods, they were both treated at the same Swiss‐level one university hospital.

### Diagnosis of PJI

The diagnosis of PJI was made according to the EBJIS criteria [30].

Acute PJI was defined as early postoperative (within 4 weeks after TKA) or acute hematogenous (symptoms less than 3 weeks in duration). Cases were excluded if they had a chronic infection (duration > 4 weeks) or if they underwent initial debridement in another hospital. Only patients with a confirmed infection were included in the final analysis [30].

### Patient characteristics

Demographic information such as age, sex, tobacco use, diabetes, intravenous drug abuse and immunomodulating medications was collected from the medical records.

### Antibiotics

For all cases, empiric broad‐spectrum intravenous antibiotic therapy—the standard being amoxicillin/clavulanic acid—was started after obtaining intraoperative tissue biopsies. In the standardized cohort, this was later replaced by targeted antibiotic therapy according to the pathogen susceptibility and recommendation of the infectious disease (ID) specialist belonging to the MDT. In the non‐standardized cohort, the ID specialist did not belong to the MDT. Antibiotic administration was transitioned from intravenous to oral therapy as soon as patients were clinically stable without systemic or local signs of uncontrolled infection, the responsible pathogen(s) were susceptible to oral antibiotics established for PJI treatment, and patients were able to take and adhere to the oral regimen [33, 38]. Following antibiotic treatment guidelines established in the PJI protocol of Zimmerli et al. [47], therapy was continued for 3 months postoperatively [6]. This did not vary between the two cohorts.

### Database

For the standardized cohort, all consecutive patients ≥18 years who underwent DAIR procedure for acute PJI after TKA between October 2019 and October 2022 were identified from our institution's prospectively collected surgical procedure database.

A total of 38 cases met the screening criteria for the standardized cohort. Further cases were excluded if (1) DAIR was performed without curative intention (*n* = 4), (2) the patient follow‐up period was less than 1 year after DAIR (*n* = 1), (3) the postoperative antibiotic therapy was independently stopped by the patient (*n* = 1) and (4) a postoperative diagnosis other than PJI was made, that is, mechanical instability (*n* = 1), crystal arthropathy (*n* = 1) or local wound healing disturbances (*n* = 4). Thus, a total of 26 cases were included in the final analysis (Figure 1).

**Figure 1:**
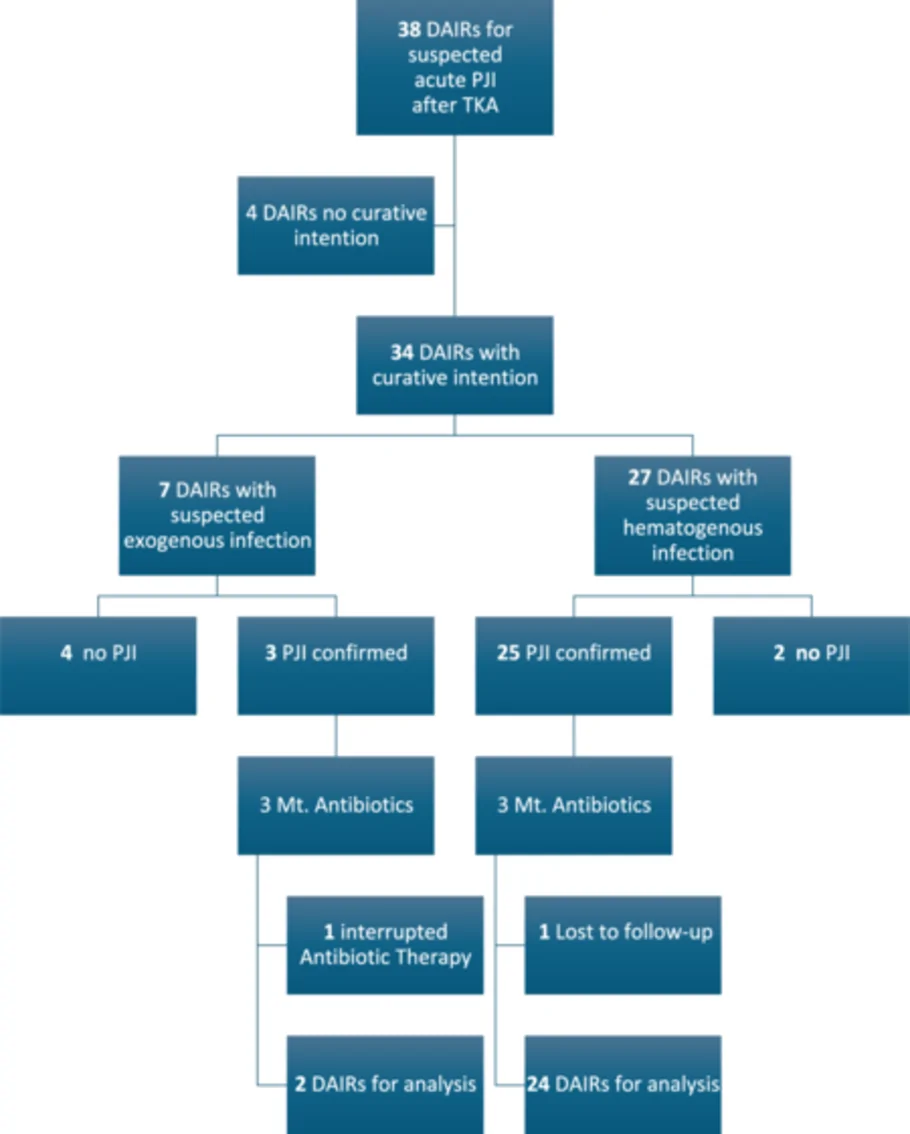
Case selection for standardized cohort. DAIRs, debridement and implant retentions; PJI, periprosthetic joint infection; TKA, total knee arthroplasty.

For the non‐standardized cohort, all patients ≥18 years who underwent DAIR for PJI after TKA between January 2005 and May 2015 at the same institution were retrospectively screened. After applying the same exclusion criteria as mentioned above, 31 patients were able to be included in the final analysis [4].

It should be noted that, in the non‐standardized cohort, the diagnosis and surgical indication were determined at the discretion of the treating surgeon, as no specialized MDT had yet been established at this time point.

### DAIR technique for the standardized cohort

All DAIRs were performed either by or under the supervision of two senior surgeons (M. M. and M. C.) belonging to the MDT, adhering to a standardized surgical technique. The incision was made through the already existing scar. Non‐vital tissue was removed. Adequate exposure was gained by fully opening the entire approach, extending proximally if needed. Arthrotomy was performed through the already existing scar tissue or after removing the remaing suture material. If not already performed during prior infection work‐up, synovial fluid was collected and sent for cell count and differentiation. The polyethylene (PE) was removed and sent for sonication. The radical synovectomy began medially and continued in a clockwise manner through the infrapatellar, lateral, posterior and suprapatellar compartments. The biopsies for histology and microbiology were consistently taken from: (1) the dorsal aspect of the extensor apparatus and retropatellar, (2) the medial, (3) lateral, (4) suprapatellar recessus and (5) posterior compartment. After haemostasis was achieved, the joint was irrigated with 1–2 L of bactericidal solution (Lavasept^®^). Afterwards, the new PE was inserted and checked for stability. The arthrotomy was closed with adaptive single suture with absorbable multifilament (Vicryl 2™; Ethicon). This was followed by a continuous subcutaneous suture (Vicryl 2‐0™; Ethicon). Skin closure was performed with either surgical skin staples or non‐absorbable suture (Ethilon 2‐0; Ethicon). A sterile wound dressing was applied and the whole leg was bandaged.

### DAIR technique for the non‐standardized cohort [4]

DAIRs were performed by board‐certified orthopaedic surgeons representing all hierarchical levels. The preexisting medial parapatellar incision was used, and non‐vital tissue was removed. An arthrotomy was performed. In contrast to the standardized technique, the radical synovectomy as well as the number and location of the tissue biopsies were done according to each surgeon's preference. If a PE exchange was performed, the explanted PE was sent for sonication. Afterward, the knee was irrigated with bactericidal solutions (Lavasept^®^). The method of arthrotomy closure, as well as the subcutaneous and skin closure were also done according to surgeon's preference.

#### Outcome

The primary outcome was treatment success in the 1‐year follow‐up period after index DAIR, defined according to the Delphi consensus criteria for successful treatment of PJI [13]. Additionally, PJI‐related mortality was defined as a complication of index or subsequent DAIR or revision procedures as per discharge notes and clinical documentation.

#### Statistical analysis

To test the primary hypothesis regarding treatment success between the standardized and non‐standardized cohorts, Fisher's exact test was applied. To compare the patient demographics between cohorts and assess baseline comparability, univariate analyses were used including independent *t* tests, Mann–Whitney *U* tests, *χ* ^2^ tests and Fisher's exact tests, depending on variable type and distribution. A significance level of *p* < 0.05 was considered statistically significant for all comparisons.

## RESULTS

In the standardized cohort, all but one patient remained infection‐free at the 1‐year follow‐up. This corresponds to a success rate of 96.3%. In comparison, six patients experienced treatment failure in the non‐standardized historical cohort, resulting in a success rate of 80.6% (*p* = 0.12).

In the standardized cohort, the single treatment failure was due to PJI‐related mortality in a patient presenting with bacteremia with *Staphylococcus aureus* and septic shock with consecutive multi‐organ failure. It should be noted that two patients in the standardized cohort required a second surgery. In the first case, the initial procedure was a life‐saving emergency intervention in a hemodynamically unstable, critically ill patient. This was aimed at reducing the bacterial load. A radical synovectomy and PE exchange could not be performed due to the patient's unstable intraoperative condition. Eight days later, once the patient was hemodynamically stable, a formal DAIR with radical synovectomy and PE exchange was performed with curative intention.

For the second case, a joint lavage was performed 12 days after the index surgery due to the development of a postoperative haematoma after initial DAIR. No additional radical synovectomy or PE exchange was performed during the joint lavage. The biopsies from the joint lavage remained sterile. Given the fact that both procedures were not performed due to infection persistence or recurrence, they were not classified as DAIR failures, in line with several other publications [1, 18, 43]. From the non‐standardized cohort, treatment failure occurred in six cases, including one of PJI‐related mortality.

Patients were significantly older (*p* = 0.02) and had a significantly longer interval from implantation to DAIR (*p* = 0.98) in the standardized cohort (Table 1).

**Table 1:** Demographic characteristics.

Variable	Standardized cohort (*n* = 26)	Non‐standardized cohort (*n* = 31)	*p* Value
Age [mean ± SD], years	75.5 ± 11.2	69.2 ± 9.7	**0.02**
Male	18 (69.2%)	16 (51.6%)	0.22
Female	8 (30.8%)	15 (48.4%)	
Tobacco use	4 (15.4%)	5 (16%)	1.00
Diabetes mellitus	9 (34.6%)	12 (39%)	0.78
Immunosuppression	2 (7.7%)	6 (19%)	0.26
Interval from TKA to DAIR (median, IQR), years	7.5 years (2.5–13.5)	1.28 years (0.4–2.8)	0.98
PJI Type: acute hematogenous	24 (92.3%)	26 (84.6%)	0.44

*Note*: Bold values highlight statistical significance.

Abbreviations: DAIR, debridement and implant retentions; IQR, interquartile range; PJI, periprosthetic joint infection; SD, standard deviation; TKA, total knee arthroplasty.

Besides the percentage of polymorph nuclear cells in synovial fluid (*p* = 0.02), no significant differences were found between the two cohorts (Table 2). *S. aureus* was the most isolated organism in both cohorts (Table 3).

**Table 2:** Preoperative laboratory values.

Variable	Standardized cohort (mean)	Non‐standardized cohort (mean)	*p* Value
CRP (mg/L, median; [IQR])	167.5 (96.5–261.0)	141 (34–197)	0.21
WBC (×10^9^/L)	11	10.3	0.42
Cell count in synovial fluid (cells/µL)	89,600	64,970	0.08
Polymorphonuclear leucocytes in synovial fluid (%)	86.3	90.6	**0.02**

*Note*: Bold values highlight statistical significance.

Abbreviations: CRP, C‐reactive protein; IQR, interquartile range; WBC, white blood cell.

**Table 3:** Microbiological culture results.

	Standardized cohort	Non‐standardized cohort	*p* Value
*Staphylococcus aureus*	8 (32.0%)	7 (24.13%)	0.55
*Streptococcus* spp.	2 (8.0%)	7 (24.13%)	0.16
Culture negative	6 (24.0%)	3 (10.34%)	0.27
*Enterococcus* spp.	1 (4.0%)	3 (10.34%)	0.61
Coagulase‐negative Staphylococci	3 (12.0%)	3 (10.34%)	1.00
Polymicrobial infection	2 (8.0%)	3 (10.34%)	1.00
Gram‐negative bacteria	3 (12.0%)	3 (10.34%)	0.68

## DISCUSSION

The treatment success of DAIR procedures is directly associated with accurate diagnostics [37], appropriate surgical technique and adequate antibiotic treatment [6]. While good evidence for diagnostics and duration of antibiotic therapy exists, the data on the impact of surgical technique are limited [9, 22, 34].

Our most important finding was the higher treatment success rate at 1‐year follow‐up in early‐ and acute hematogenous PJI after TKA when following a standardized DAIR protocol performed by surgeons belonging to an MDT compared to a recently published historic cohort from the same institution [4]. Even though no statistical difference was observed, the treatment success rate of 96.3% was higher than that observed in the non‐standardized cohort. It was also above the upper range of what most publications report, usually around 60%–88% [2, 5, 8, 10, 11, 23, 24, 28, 29, 34, 39].

Although studies addressing the impact of the DAIR technique on clinical outcomes are scarce, this finding is supported by similar publications. In a prospective multicenter cohort study, an 84% infection‐free survival rate at 2 years was reported by Karlsen et al. [22], while Brivio reported an 84% success rate in the setting of knee hemiarthroplasty [9]. Both followed a protocolized DAIR technique with concomitant MDT support.

Even though the basic principles for managing implant‐associated infections were established more than 20 years ago [46, 47] and good outcomes for PJI following TKA and total hip arthroplasty have been achieved [17, 26, 42], a series of punctual interventions since then have changed the paradigms of treatment. Starting with establishing the diagnosis, Sousa et al. recently showed how an improved diagnostic sensitivity using the EBJIS definition enabled earlier intervention, particularly in cases that would have been missed following criteria from other societies [37]. Furthermore, Sendi et al. demonstrated that algorithm‐guided diagnostics, patient selection and standardized treatment by dedicated surgeons resulted in infection control in 93% of cases after PJI of the hip [34]. These findings are supported by other studies showing improved clinical outcomes when a MDT is involved early on [7, 15, 44]. This, in turn, results in protocolized diagnostic and treatment algorithms, improved decision making regarding the choice of surgical strategy and a standardized DAIR technique. Additionally, the consistent performance of open revision arthroplasty procedures during daytime hours by or under the supervision of two senior surgeons (M. M. and M. C.) belonging to the MDT could have also positively impacted our results.

In both cohorts, one patient died after index DAIR due to complications related to uncontrollable infection. Literature regarding mortality after DAIR is scarce. Urish et al. reported a 5‐year mortality rate of 19.9% [40]. In a study involving 34 patients, synchronous PJI was associated with a 30‐day mortality of 18% [16]. Similarly, a high mortality rate after PJI treatment for non‐infectious reasons has been documented [31].

The strength of this study is the uniform definition for PJI and the antibiotic treatment for both cohorts, thus reducing the risk of selection bias. Additionally, both cohorts were observed to be demographically comparable.

This study has several limitations. Firstly, due to the small sample size, the obtained results are statistically underpowered. Secondly, there was a different follow‐up time between the two cohorts. Given that most treatment failures occur within 1 year after index DAIR [12, 32], the treatment success was determined at this time point for the standardized cohort. For the non‐standardized cohort, the follow‐up was longer, with a mean of 8.4 years. Thus, theoretically allowing for late onset of failure. Thirdly, the retrospective design may introduce selection and information biases. Lastly, the single‐centre setting limits the generalizability of the findings to other institutions with different patient populations and clinical practices.

The findings of this study can relevantly impact daily clinical practice by offering a structured and reproducible DAIR technique that can be consistently applied by surgical teams to improve the management of acute PJI after TKA. Additionally, it provides supporting evidence for the implementation of a dedicated MDT for the management of PJI.

## CONCLUSIONS

This study highlights that although statistical significance was not reached, adhering to a standardized DAIR technique performed by surgeons belonging to a dedicated MDT increases treatment success rates in acute PJI of the knee. To further identify the real magnitude of the observed clinical trend and the duration of the infection‐free interval, more rigorous and longer prospective study designs are needed. Nevertheless, the implementation of such a multidisciplinary and protocolized treatment approach, as utilized in our institution, can only be beneficial for patients undergoing this severe complication.

## AUTHOR CONTRIBUTIONS

Guillermo Sánchez‐Rosenberg performed data collection and analysis, interpreted the results and drafted the manuscript. Martin Clauss conceptualized the study. Mario Morgenstern and Martin Clauss either performed or supervised the DAIRs included in the study. Anna Bartsch performed data collection and reviewed the manuscript. Mario Morgenstern, Richard Kuehl and Martin Clauss reviewed and approved the final version of the manuscript.

## CONFLICT OF INTEREST STATEMENT

The authors declare no conflicts of interest.

## ETHICS STATEMENT

This project was granted approval by the local ethics committee, the Ethical Commission of Northwest and Central Switzerland, under the disposition EKNZ 2023‐01960.

## Data Availability

The data that support the findings of this study are not available due to patient data privacy concerns.
